# Entropic Inhibition: How the Activity of a AAA+ Machine Is Modulated by Its Substrate-Binding Domain

**DOI:** 10.1021/acschembio.1c00156

**Published:** 2021-03-19

**Authors:** Marija Iljina, Hisham Mazal, Pierre Goloubinoff, Inbal Riven, Gilad Haran

**Affiliations:** † Department of Chemical and Biological Physics, Weizmann Institute of Science, Rehovot 761001, Israel; ‡ Department of Plant Molecular Biology, Faculty of Biology and Medicine, University of Lausanne, CH-1015 Lausanne, Switzerland

## Abstract

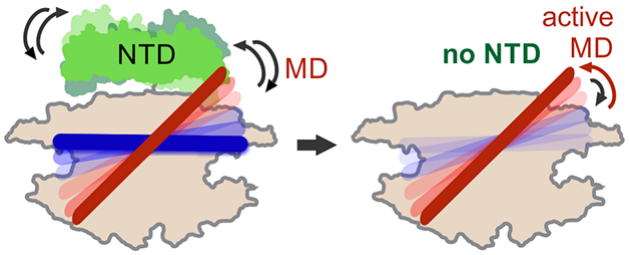

ClpB is a tightly regulated AAA+ disaggregation machine. Each ClpB molecule is composed of a flexibly attached N-terminal domain (NTD), an essential middle domain (MD) that activates the machine by tilting, and two nucleotide-binding domains. The NTD is not well-characterized structurally and is commonly considered to serve as a dispensable substrate-binding domain. Here, we use single-molecule FRET spectroscopy to directly monitor the real-time dynamics of ClpB’s NTD and reveal its unexpected autoinhibitory function. We find that the NTD fluctuates on the microsecond time scale, and these dynamics result in steric hindrance that limits the conformational space of the MD to restrict its tilting. This leads to significantly inhibited ATPase and disaggregation activities of ClpB, an effect that is alleviated upon binding of a substrate protein or the cochaperone DnaK. This entropic inhibition mechanism, which is mediated by ultrafast motions of the NTD and is not dependent on any strong interactions, might be common in related ATP-dependent proteases and other multidomain proteins to ensure their fast and reversible activation.

N-terminal domains (NTDs) tune the functions of numerous proteins by autoinhibitory intramolecular interactions with cognate domains^1^ and by propagating allosteric signals to remote sites through disordered linkers.^2^ Examples include control of gating in the neurotransmitter receptor NMDA upon binding of allosteric drugs to the NTD,^3^ activation of the regulation of the unfoldase ClpC upon binding of its NTD to the adaptor protein MecA,^4^ control of proteolytic activity of the 20S core-particle proteasome by fast conformational switching of NTD residues,^5^ or suppression of the protease ClpP due to the unfolding of its NTD.^6^ NTD-bearing AAA+ proteins (ATPases associated with diverse cellular activities) are large ATP-dependent molecular machines^7^ with a diverse set of tightly regulated functions.^8^ The bacterial heat-shock protein ClpB is an AAA+ machine that works in collaboration with the DnaK chaperone system (DnaK, DnaJ, and GrpE) to rescue proteins from aggregates and is essential for conferring thermotolerance.^9,10^ In its functional form, ClpB is a ring-shaped homohexamer that disaggregates substrate proteins by actively threading them through its central channel in an ATP-dependent process.^11^ Each monomer of ClpB comprises several domains^12^ (Figure 1a): a flexibly connected NTD, two ATP-binding domains (NBD1 and NBD2), and the regulatory middle domain (MD) that activates the machine by ultrafast toggling.^13^ The NTD is a globular, 135–147-residue α-helical domain, with a structure that is highly conserved across the ClpA, ClpB, and ClpC subfamilies^14^ and in eukaryotic homologues.^15^ In bacteria, a truncated variant of ClpB lacking the NTD, ΔNClpB, is naturally coexpressed as a minor product together with the full-length protein^16,17^ and was reported to contribute significantly to bacterial thermotolerance.^18^ The biological relevance of the truncated variant, compared to the full-length protein, is not clear.

**Figure 1:**
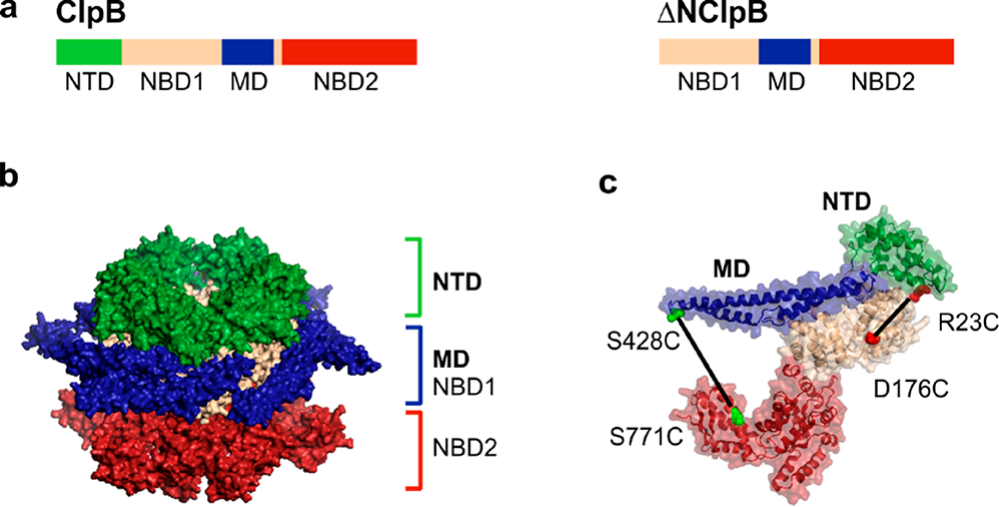
Structure of ClpB. (a) The sequence of domains in the full-length ClpB. A truncated, naturally occurring variant of ClpB, ΔNClpB, lacks the NTD. (b) Structure of the ClpB hexamer with the NTDs highlighted in green (PDB: 1QVR).^12,30^ (c) Structure of the ClpB monomer with the NTD shown in green and the MD in blue. Pairs of residues mutated to cysteines for the incorporation of fluorescent dyes are highlighted: R23C and D176C (in red) for the characterization of NTD dynamics; S428C and S771 (in green) for the measurement of MD dynamics.

The six NTDs of ClpB form a distinct large ring at the top of the hexamer (Figure 1b).^12^ These domains serve as initial binding sites for certain substrate–proteins^19,20^ prior to their processing. Because of its high mobility, the entire NTD ring is frequently unobserved in cryo-electron microscopy (cryo-EM) reconstructions of ClpB hexamers.^21−24^ However, two recent cryo-EM studies employed “focused classification” to capture several substrate-bound NTDs.^25,26^ They revealed NTD trimers that made contacts with substrate protein molecules and surrounded the central channel of ClpB. In addition, a full ring of six NTDs was resolved in a cryo-EM structure of the yeast homologue of ClpB, Hsp104, also showing that they interact with the substrate.^27^ The substrate-binding properties of the NTDs were also characterized in several biochemical studies,^19,28,29^ and the substrate-binding groove was mapped by NMR spectroscopy.^20^

Although the experimentally validated function of the NTDs in ClpB remains limited to substrate–protein engagement, accumulating evidence suggests that their role might be more complex. Several studies reported an increased ATP hydrolysis rate by the NTD-truncated mutant ΔNClpB in the absence of protein substrates, compared to the full-length ClpB, indicating that the NTDs may inhibit futile ATP hydrolysis through yet uncharacterized communication pathways.^17,19,20,31−36^ Indeed, potentially relevant interactions of the NTD with the MD and NBDs in solution were recently detected by X-ray footprinting in Hsp104.^37^ Furthermore, the function of the NTDs during substrate processing and translocation remains uncertain. Recently, an NTD deletion was reported to abolish the translocation of maltose-binding protein.^38^ On the other hand, ΔNClpB remained active in multiple *in vitro* disaggregation assays in the presence of DnaK-DnaJ-GrpE with other preaggregated protein substrates, implying a secondary role for the NTD in this process.^20,28,29,32,39^ It was proposed that the NTDs block the central channel^20^ and can undergo conformational changes to actively assist with the translocation and disaggregation of substrate–proteins.^29^ NMR spectroscopy in aqueous solution suggested fast movement of the NTDs.^20^ These dynamics have not been studied in detail and the time scales of the motion, any accompanying conformational rearrangements, and their implications for the machine function remain to be characterized.

To shed light on the equivocal roles of the NTDs in ClpB, we have used a combination of single-molecule FRET (smFRET) spectroscopy and biochemical experiments. We directly monitor the submillisecond motions of the NTDs and demonstrate that engagement of substrate proteins does not restrict these conformational dynamics. We characterize the communication of the NTD with other functional domains of ClpB and find that the ultrafast motions of the NTD restrict sterically the MD conformations, thus maintaining the inactive state of the MD. This mechanism, which we term *entropic inhibition*, results in suppressed ATPase and disaggregation activities of ClpB. Our results suggest that the NTD is critically involved in the reversible autoinhibition of ClpB through its ultrafast dynamics.

## Results

### The NTD Fluctuates on the Microsecond Time Scale with Changes in Conformational Dynamics upon Substrate Binding

In the crystal structure of the full-length *Thermus thermophilus* (*TT*) ClpB (henceforth ClpB),^12^ the NTDs are connected to the neighboring NBD1 domains by disordered linkers, which might suggest that these domains are highly mobile. Analysis of the NTD dynamics in a related AAA+ hexameric machine p97 by solution NMR spectroscopy suggested microsecond motions.^40^ Here, we set to characterize the real-time dynamics of the NTD within the ClpB hexamer in aqueous solution. As in our recent study of the MD dynamics in the full-length ClpB,^13^ we employed a combination of smFRET and a maximum likelihood analysis method (H^2^MM), described in detail previously.^41^ Briefly, H^2^MM is a hidden Markov model technique that analyzes smFRET data on the level of individual photons in order to extract fast dynamics. To study the NTD dynamics, we located a cysteine for fluorescent labeling on the NTD, at the end of α-helix A1 (residue R23), and a second cysteine was inserted into a rigid position within NBD1 (residue D176) of ClpB (Figure 1c). The resulting double mutant of ClpB was labeled with Alexa Fluor 488 (AF488) and Alexa Fluor 594 (AF594; Methods). It was confirmed that the fluorescently labeled 23C-176C construct exhibited unaltered ATPase activity (3.2 ± 0.1 min^–1^ relative to 3.5 ± 0.2 min^–1^ in WT ClpB).

**Figure 2:**
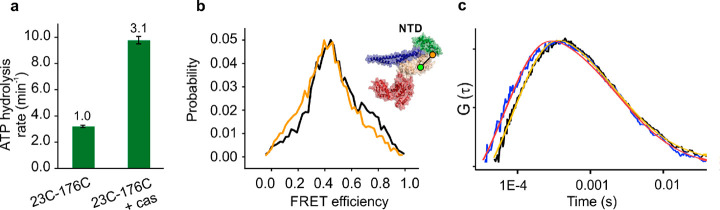
Analysis of the conformational dynamics of the NTD. (a) ATPase activity of fully labeled 23C-176C protein, either basal or in the presence of 25 μM κ-casein (*n* = 3). The error bars correspond to standard errors of the mean. The numbers above the bars designate relative activity. (b) FRET efficiency histograms of the 23C-176C mutant of ClpB with a single labeled protomer in a 2 mM ATP solution, either in the absence of substrate–protein (black) or in the presence of 25 μM κ-casein (yellow). Here and elsewhere, FRET efficiency histograms are area-normalized. (c) Filtered FCS cross-correlation curves of 23C-176C ClpB without (black) and with 25 μM κ-casein (blue). The red and yellow curves are fits to eq 1 in the Methods and yield similar diffusion coefficients but faster dynamics in the presence of κ-casein (red) than in its absence (yellow)—see text.

The labeled protein showed prominent ATPase stimulation upon binding of the model protein substrate κ-casein^42^ (3.1-fold enhancement, Figure 2a). We have shown before that ClpB hexamers are stable and demonstrate no assembly defects under our experimental conditions, using cryo-EM and interprotomer smFRET experiments.^13^ Here, we used native gel electrophoresis to demonstrate that the 23C-176C construct had no assembly defects in the presence of 2 mM ATP (Figure S1). Furthermore, fluorescence anisotropy measurements of single-cysteine mutants (23C and 176C) indicated that the dyes attached at these positions showed unrestricted rotation that was not affected by the presence of κ-casein (Figure S1).

Double-labeled ClpB subunits were mixed with a 100-fold molar excess of unmodified unlabeled ClpB subunits (WT ClpB), ensuring that the newly assembled ClpB hexamers contained only one fluorescently labeled protomer. We conducted photon-by-photon smFRET measurements on diffusing molecules of the construct introduced above in the presence of 2 mM ATP. The arrival times of the photons emitted by both donor (AF488) and acceptor (AF594) dyes were registered on two separate detectors. After selecting computationally molecules that contained both AF488 and AF594 dyes (Methods and Figure S4), we calculated FRET efficiency histograms. The histograms were broad (Figure 2b), indicating multiple conformations of the NTD. Surprisingly, there was a significant shift to lower FRET efficiency values in the presence of κ-casein, meaning that longer distances between residues 23C and 176C became more populated. This indicates that the NTD’s equilibrium positions become closer to the central channel, in agreement with previous proposals that the NTDs direct bound substrate–proteins toward the central pore.^29^ We made sure that the shift of the FRET efficiency histograms was not due to photophysical effects by comparing properties of fluorescence bursts in experiments with and without κ-casein (Table S1). In a control experiment, we relocated one dye from the NTD to NBD2 (739C), and the FRET efficiency histograms of the mutant 176C-739C were identical with and without κ-casein (Figure S1), supporting the lack of unwanted photophysical effects.

To assess the time scale of the NTD motion within ClpB hexamers, we analyzed the dynamics by fluorescence lifetime correlation spectroscopy (FLCS). This experimental technique^43^ enables filtering fluorescence cross-correlation functions to obtain the contribution of separate subspecies observed in the FRET histogram (see Methods). We calculated cross-correlation functions between the range of species in the histograms of Figure 2b with the lowest FRET efficiency values (<0.18) and the range of species with the highest FRET efficiency values (>0.75), to obtain the overall exchange rate between these population ranges. The resulting curves (Figure 2c) showed a prominent rise of the signal on the microsecond time scale, and analysis indicated conformational dynamics with an exchange rate of 8330 ± 630 s^–1^. Interestingly, the addition of κ-casein at a concentration of 25 μM led to acceleration of the dynamics to 12 500 ± 780 s^–1^. The reasons for the observed increase in the NTD dynamics with κ-casein elude us at this point in time (see Discussion).

For a more quantitative analysis of the various states occupied by the NTD, we applied H^2^MM to individual smFRET photon trajectories,^41^ using a model with three states, which was the smallest number of states required to properly fit the smFRET data (see below). From this analysis, we could learn about the states in the system and the rates of their interconversion. The H^2^MM-derived FRET efficiency values were 0.18, 0.49, and 0.81 for states 1, 2 and 3, correspondingly, and the transition rates out of these states were 3540 ± 480 s^–1^, 1860 ± 150 s^–1^, and 2500 ± 220 s^–1^, respectively, and were increased in the presence of κ-casein (Figure S1, Table S2), consistent with the FLCS analysis. We compared the H^2^MM-derived FRET efficiency states to the theoretical values based on available ClpB structures (Supplementary Methods and Table S3). From this analysis, state 1 at a FRET efficiency of 0.18 was consistent with the NTD conformation captured in the cryo-EM structure of substrate-unbound ClpB where this domain plugs the central channel,^25^ and state 3 at 0.81 was in agreement with the NTD conformation found in the crystal structure of ClpB.^12^

To validate these results, we performed an analysis of dwell time distributions,^13,44^ and the resulting escape rates were in close agreement with the values obtained from H^2^MM (Figure S1, Table S2), suggesting that three states fit the data well. From the H^2^MM-derived FRET efficiency values of states 1 and 3 (0.18 and 0.81), we calculated the amplitude of the NTD motion to be ∼28 Å. Together, these results show that, within the hexamer, the NTD undergoes ultrafast large-scale movements that are independent of nucleotide state and are only mildly accelerated by the binding of the model substrate–protein. The high speed and conformational freedom are likely to underlie all of the NTD-driven regulations in ClpB that we characterize in subsequent sections.

### The NTD Suppresses ATPase and Disaggregation Activities of ClpB

**Figure 3:**
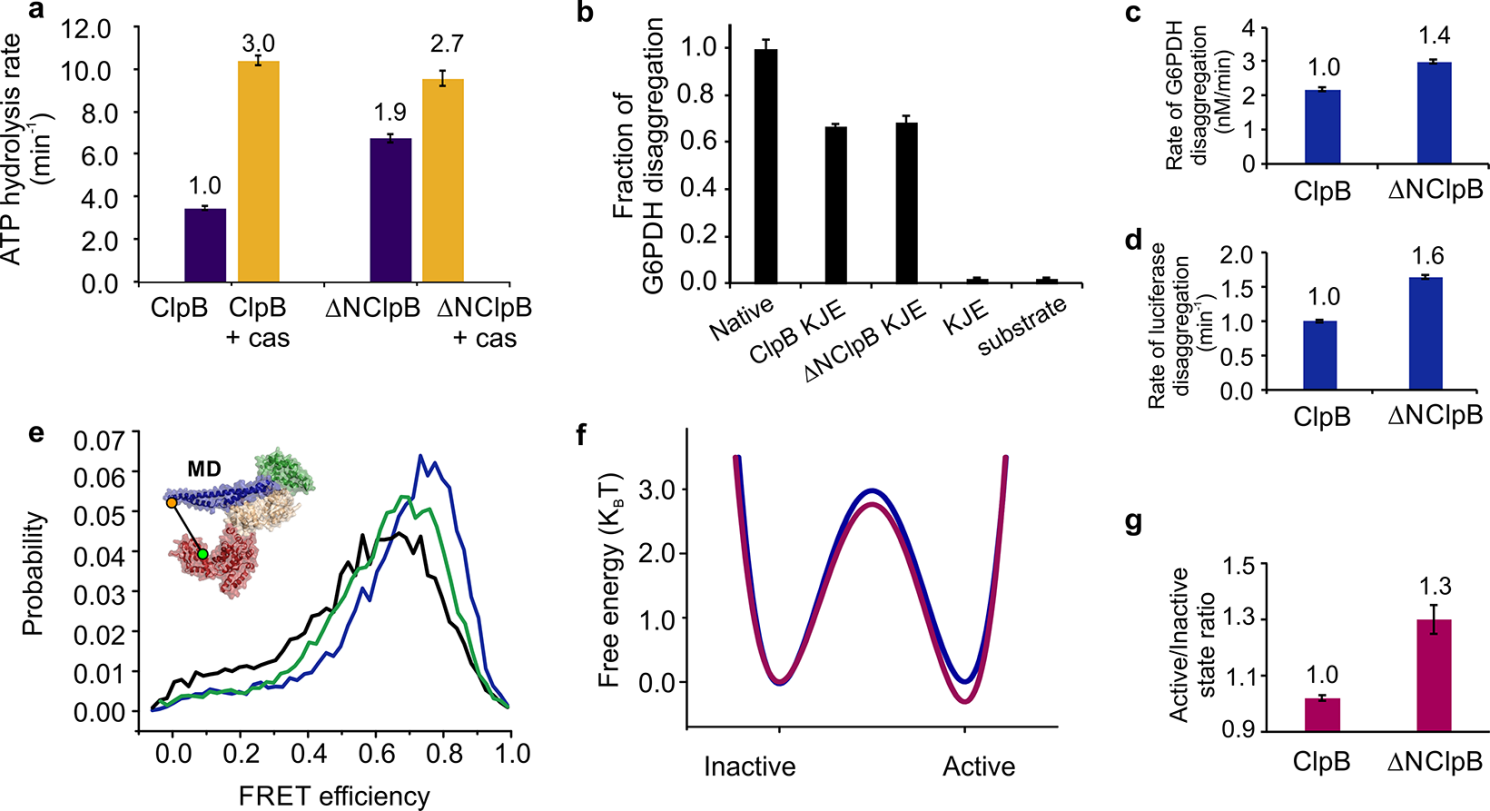
ATPase, disaggregation activities, and MD dynamics of ClpB and ΔNClpB. (a) Rate of ATP hydrolysis measured at 25 °C with and without 50 μM κ-casein (“cas,” *n* = 3). (b) Disaggregation of heat-induced aggregates of G6PDH as a substrate, measured after 3 h of incubation with ClpB variants (2 μM) in the presence of DnaK, DnaJ, and GrpE (2 μM, 1 μM, and 1 μM, respectively). The fraction of disaggregation was determined by taking the activity of native (nonaggregated) G6PDH as 1 (labeled “Native”, *n* = 6). (c,d) Rate of disaggregation of heat-induced aggregates of G6PDH (c) or of firefly luciferase (d; see disaggregation yields in the main text) by ClpB variants in the presence of DnaK, DnaJ, and GrpE (*n* = 3). (e) FRET efficiency histograms of the full-length S428C–S771C construct of ClpB (black) and of the respective ΔNClpB mutant in the presence of 2 mM ATP (green) and in the presence of 25 μM κ-casein (blue). (f) Comparison of the free-energy profiles of MD dynamics of the full-length ClpB (blue) and ΔNClpB (purple), calculated from H^2^MM parameters using the Arrhenius equation with a pre-exponential factor of 10^5^ s^–1^. (g) The population ratio of horizontal (inactive) to tilted (active) states of the MD, derived from H^2^MM analysis. The error bars in all panels correspond to standard errors of the mean. In a, c, d, and e, values above the bars are the fold increases relative to the corresponding values for ClpB (which are set to 1).

To further understand the role of the NTD in ClpB’s activity, we expressed a truncated version, ΔNClpB, which lacks the first 140 residues of the protein (Figure S3). We confirmed by native polyacrylamide gel electrophoresis and by size-exclusion chromatography that both ClpB and ΔNClpB were well-assembled (Figure S2). Measurements of ATP hydrolysis rates in the presence of 2 mM ATP at 25 °C yielded a doubled value for ΔNClpB compared to ClpB (6.8 ± 0.3 min^–1^ vs 3.5 ± 0.2 min^–1^; Figure 3a), in close agreement with previous literature values.^13,32,45^

However, in the presence of κ-casein (50 μM), similar maximal hydrolysis rates were reached by ClpB and ΔNClpB (10.4 ± 0.4 min^–1^ and 9.6 ± 0.6 min^–1^, respectively, Figure 3a). The results suggest that the truncated ClpB variant is dysregulated and exhibits futile ATPase activity in the absence of protein substrate. We compared the disaggregation activity of ClpB and ΔNClpB by monitoring the reactivation of heat-induced aggregates of glucose-6-phosphate dehydrogenase (G6PDH)^46^ and observed a ∼67% regeneration of soluble G6PDH activity by both ClpB and ΔNClpB (Figure 3b). Even though the yields of disaggregation by the two variants were essentially the same, ΔNClpB displayed a higher disaggregation rate compared to the full-length variant (3.0 ± 0.1 nM min^–1^ vs 2.2 ± 0.1 nM min^–1^, Figure 3c). This difference in the rates of disaggregation was also observed in experiments with heat-induced aggregates of firefly luciferase (21.7 ± 0.5 min^–1^ for ΔNClpB compared to 13.1 ± 0.65 min^–1^ for ClpB; Figure 3d), in which the final yields of luciferase regeneration were 43 ± 3% for ClpB and 76 ± 5% for ΔNClpB.

### smFRET Experiments Reveal That ΔNClpB Has Activated MDs

To shed light on the effect of the NTD deletion on ClpB activity, we probed the deletion influence on MD dynamics by using smFRET measurements and H^2^MM analysis.^13,41^ In a recent study, we reported that the MDs of ClpB are highly dynamic, moving on the submillisecond time scale between their collinear (inactive) and tilted (active) states.^13^ Our results indicated that the MD is a continuous (analog), rather than a two-state (digital), activation switch for ClpB, with the population ratio of its states affecting the overall activity of the machine. Strikingly, in the absence of substrates or cochaperones, the two states in the full-length ClpB were populated equally, with an active/inactive population ratio of 1.00 ± 0.01, and were rapidly interconverting with transition rates of *k*_12_ = 5300 ± 150 s^–1^ and *k*_21_ = 5700 ± 100 s^–1^, respectively. This population ratio was shown to be significantly modulated by a range of allosteric signals, such as the binding of DnaK, nucleotides, and substrate proteins.

To characterize the MD dynamics of ΔNClpB by smFRET, we generated a double-cysteine mutant of ΔNClpB (Figure 1c). The first cysteine was introduced into motif 1 of the MD (residue 288, which corresponds to 428 in the full-length ClpB) and the second into NBD2 (residue 631, corresponding to 771 in the full-length ClpB, Figure 1c). The resulting double mutant of ΔNClpB was labeled with AF488 and AF594 (Methods) and mixed with a 100-fold molar excess of unmodified ΔNClpB to obtain a single fluorescently labeled protomer within each hexamer. We tested the effect of labeling on ΔNClpB, and found no change in ATPase or disaggregation activity (Figure S2), in agreement with previous findings on the full-length ClpB.^13^ smFRET measurements were carried out on ΔNClpB in solution, in the presence of 2 mM ATP. The addition of ATP guaranteed the stability of ClpB hexamers in all our smFRET experiments;^13^ indeed, FRET efficiency histograms showed no evidence for their dissociation during the measurements (Figure S5).

FRET efficiency histograms of ΔNClpB appeared to be shifted to higher average FRET efficiency values compared to the data for the full-length ClpB (Figure 3e). The two major states of the MD were found to possess FRET efficiency values of 0.8 ± 0.01 (state 1) and 0.48 ± 0.01 (state 2), in good agreement with the active and inactive states of the full-length variant.^13^ In contrast to the results for the full-length ClpB, though, the states were unequally populated, with relative populations of 0.56 ± 0.02 and 0.44 ± 0.02, respectively, and an increased active/inactive state ratio of 1.30 ± 0.05 instead of 1.00 ± 0.01 (Figure 3g), commensurate with the overall shift of the FRET histogram to higher value. As in the full-length ClpB, the two states were found to be under fast exchange, with interconversion rates of *k*_12_ = 4800 ± 200 s^–1^ (from active to inactive state) and *k*_21_ = 6300 ± 90 s^–1^ (reverse direction; Table S5). Approximate free energy profiles of the MD dynamics, constructed based on the derived rates, showed that the energy barrier for the transition from inactive to active state was decreased in ΔNClpB, and the active state was stabilized (Figure 3f). These results indicate that the MD in the ΔNClpB variant toggles between the same conformations as in the full-length variant but, remarkably, favors the active conformation. On the basis of our previous findings, the higher active/inactive state ratio should lead to a larger disaggregation rate of ΔNClpB,^13^ as is indeed observed (Figure 3c,d). To find whether this activation of the MD in ΔNClpB could be enhanced further by protein substrates, we repeated the experiments in the presence of 25 μM κ-casein. The resulting FRET efficiency histograms showed higher average FRET efficiency values (Figure 3e, Tables S4 and S5) due to a further increase in the active/inactive state ratio to 1.76 ± 0.01, indicating that additional activation of the MD in ΔNClpB can take place in response to substrate binding.

### The NTD Suppresses the MD through Direct Contacts

Next, we set out to explain why the MD appears activated upon the deletion of the NTD. It was previously found biochemically that the isolated NTD of ClpB from *Escherichia coli* (*E. coli)* does not form stable contacts with other parts of the molecule,^47^ and we confirmed this result for *TT* ClpB, used in this study. ΔNClpB and the isolated NTD, comprising residues 1–141 of ClpB, that were mixed together (at 40 μM ΔNClpB and 650 μM NTD) migrated separately in size exclusion chromatography and native polyacrylamide gel electrophoresis analysis (Figure S6), indicating that no stable complex was formed.

**Figure 4:**
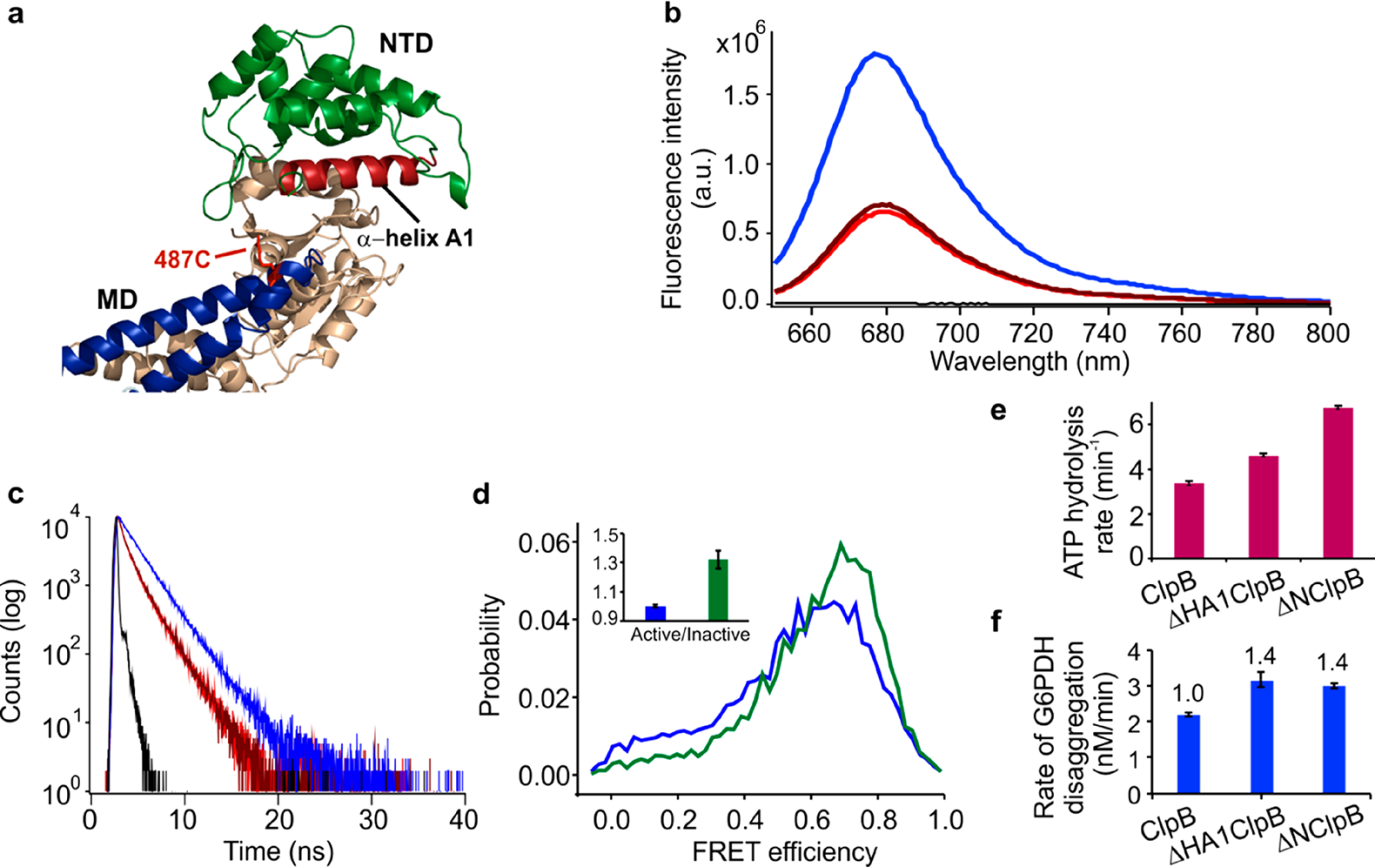
NTD mutants of ClpB affect MD dynamics. (a) Zoom into the upper region of ClpB protomer (crystal structure PDB: 1QVR).^12^ The MD is colored in blue, and the NTD in green, with α-helix A1 of the NTD highlighted in red. Residue 487C on the MD used for Atto 655 incorporation is shown as red sticks. (b) Steady-state fluorescence spectra of Atto 655-labeled ClpB mutants: ClpB (blue), 12W-ClpB (red), 23W-ClpB (dark red), buffer (black). Average fractions of fluorescence intensity, normalized to the result of the mutant without incorporated tryptophan, were 0.36 for 12W and 0.39 for 23W. (c) Fluorescence decay curves: ClpB (blue), 12W-ClpB (red), 23W-ClpB (dark-red), instrument response function (black). The resulting fluorescence lifetimes were 2 ns for the control ClpB mutant and 1.3 ns for 12W and 23W variants. (d) FRET efficiency histograms of the full-length ClpB (blue) and the truncated ΔHA1ClpB variant (green). Inset shows the corresponding active/inactive state ratios of the MD. (e) ATPase activity of ΔHA1ClpB, compared to the full-length and ΔNClpB variants (*n* = 4). (f) Rate of disaggregation of G6PDH aggregates in the presence of DnaK, DnaJ, and GrpE (2 μM, 1 μM, and 1 μM, respectively, *n* = 3). The error bars correspond to standard errors of the mean.

Since the NTD is connected to the neighboring NBD1 by a flexible linker region,^12^ it is interesting to ask whether its rapid motions in aqueous solution allow it to reach the MD. We first qualitatively assessed this possibility by using a simple computational procedure that sampled the conformational space of the NTD (see Methods section). From the inspection of the crystal structure of ClpB,^12^ we found that the region of the MD that is closest to the NTD is the edge of its motif 2. Starting with the crystal structure of the ClpB monomer,^12^ we generated multiple conformations of the NTD within the context of the full hexamer by fully rotating it around a single residue in the flexible linker region (position 142). Then, we selected NTD residues that were located within 10 Å from the edge of motif 2 (residue 487) in the generated conformations (Figure 4a) and found 11 in total, five of which were on or close to α-helix A1 of the NTD (these residues are indicated in Figure S3). On the basis of this finding, we designed ClpB mutants in order to test whether we can experimentally detect an interaction between α-helix A1 and the MD. To this end, we used Atto 655, a fluorophore that is effectively quenched upon the interaction with several amino acids, especially with tryptophan (W), leading to a significant decrease in fluorescence signal.^48^ We introduced a cysteine into the MD (position 487) of the full-length ClpB for labeling with maleimide-linked Atto 655. We then incorporated single tryptophan residues into the NTD, either at position 12 or 23 within α-helix A1 (Figure S3), and used a mutant without these additional tryptophans as a control. Fully labeled ClpB mutants Atto 655–487C-ClpB, Atto 655–487C-12W-ClpB, and Atto 655–487C-23W-ClpB were found to be well-assembled in the presence of 2 mM ATP and showed ATPase activity that was comparable to that of unmodified ClpB. 487C-ClpB variants were fully active in disaggregation experiments before labeling but had no disaggregation activity when fully labeled, likely due to the hindrance of the DnaK binding site^49^ on the MD by Atto 655 (Figure S7).

Bulk steady-state fluorescence measurements of the Atto 655-labeled variants (Figure 4b) showed a strong decrease (around 60%) of the fluorescence intensity in the tryptophan-containing mutants relative to the control 487C-ClpB, and the fluorescence lifetimes of these variants were found to be shortened (from 2 ns in the control samples to 1.3 ns in 12W- and 23W-containing variants). These differences indicate quenching of the fluorophore by the tryptophan residues in the NTD. Since the fractional decrease in fluorescence intensity of the mutants was larger than the decrease in fluorescence lifetime, we concluded that the quenching process has both a static component, due to the formation of a nonfluorescent Atto 655-quencher complex, and a dynamic component, due to transient encounters of the fluorophore with the quencher.^50^ This finding is commensurate with previous results on the effect of tryptophan on Atto 655 fluorescence.^48^ Considering that the dye is located on the tip of the MD, these results are in agreement with our hypothesis that the NTD can make contacts with the MD.

In order to determine whether the contacts that the NTD makes with the MD are sufficient to affect the MD dynamics, we removed the entire α-helix A1 from the NTD by deleting residues 8–25 of the full-length ClpB (Figure 4a, Figure S3). This truncated mutant (ΔHA1ClpB) was fully assembled (Figure S7), and smFRET analysis of its double-labeled variant revealed that its MD was activated in comparison with the MD of the full-length ClpB. Indeed, FRET efficiency histograms (Figure 4d) were shifted to higher FRET values when compared with the histograms for the full-length ClpB. The derived interconversion rates of the MD were unequal, *k*_12_ = 4400 ± 100 s^–1^ and *k*_21_ = 5900 ± 300 s^–1^ (Table S5), resulting in an increased active/inactive state ratio of 1.32 ± 0.06, comparable to the ratio found in ΔNClpB (Table S4). Measurements of the ATP hydrolysis rate of ΔHA1ClpB yielded a value of 4.6 ± 0.1 min^–1^ (Figure 4e), intermediate between the activity of the full-length ClpB (3.5 min^–1^) and ΔNClpB (6.8 min^–1^). The rate of disaggregation of heat-treated G6PDH by ΔHA1ClpB was higher than by the full-length ClpB (3.2 ± 0.2 nM min^–1^ vs 2.2 ± 0.1 nM min^–1^, Figure 4f). Therefore, removal of the interactions with α-helix A1 of the NTD activates the MD. This is consistent with the fluorescence quenching results, which indicate a direct contact of α-helix A1 with the MD. These results imply that α-helix A1 comprises residues that can reach the MD and significantly affect its conformational transitions.

### NTD Removal Does Not Disrupt Communication between the MD and NBDs

**Figure 5:**
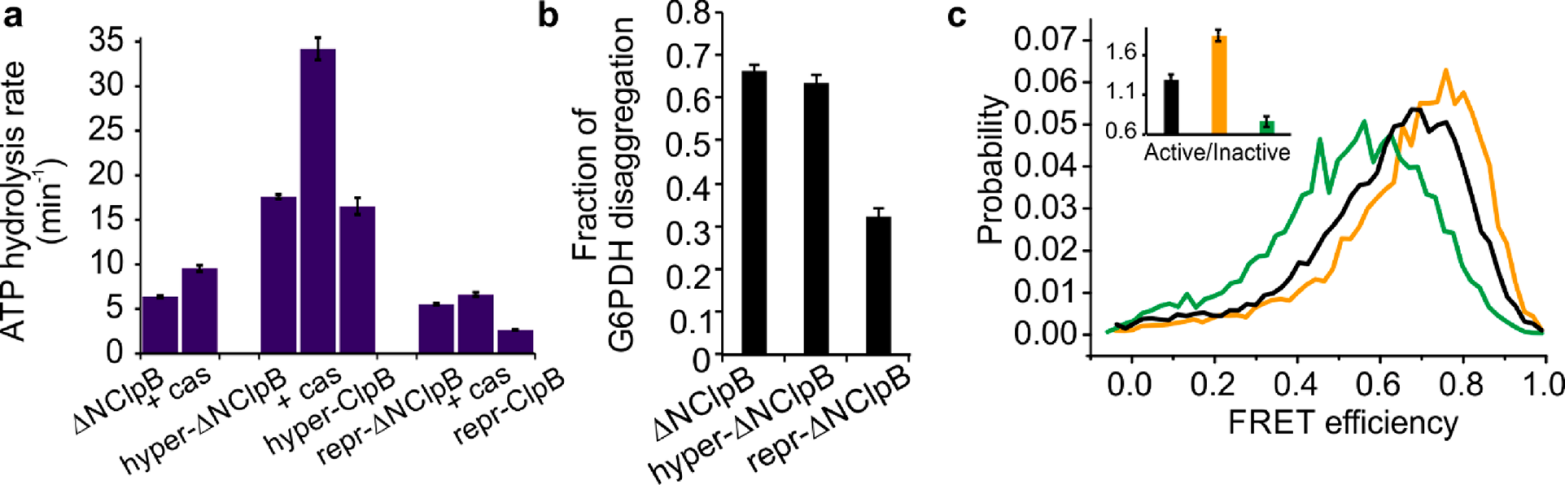
Characterization of MD-NBDs allosteric pathways in ΔNClpB. (a) The rate of ATP hydrolysis of activated and repressed ΔNClpB mutants at 25 °C with and without (*n* = 3–5) 25 μM κ-casein. The basal rates of full-length hyper- and repr-ClpB are included for reference.^13^ (b) Disaggregation yield of heat-induced aggregates of G6PDH by ΔNClpB mutants, measured after 3 h of disaggregation in the presence of DnaK, DnaJ, and GrpE (2 μM, 1 μM, and 1 μM, respectively, *n* = 2). (c) FRET efficiency histograms of activated and repressed mutants of ΔNClpB: ΔNClpB (black), hyper-ΔNClpB (orange), repr-ΔNClpB (green). Inset shows the corresponding active/inactive state ratios of the MD. The error bars correspond to standard errors of the mean.

Several mutations that are located remotely from the NTD or the NTD-NBD1 linker have been shown to promote or repress the activity of ClpB.^22,51,52^ We asked whether these mutations still have the same effect in ClpB, after its NTD is deleted. The MD is known to be tightly regulated through a network of salt bridges that connect it to the neighboring NBD1.^22,51,52^ To check whether this regulatory pathway is retained upon the NTD deletion, we generated activated and repressed hexamers of ΔNClpB by introducing previously characterized single mutations that were expected to alter the MD dynamics (Figure S3). Point-mutation E209A in NBD1 (termed hyper-ΔNClpB, corresponding to E349A in the full-length ClpB) breaks a salt bridge connecting it to NBD1, which results in a detached and activated MD.^52^ The hyper-ΔNClpB variant showed 3.5-fold higher rate of ATP hydrolysis than the unmodified ΔNClpB, an increased disaggregation rate (3.3 ± 0.1 nM min^–1^), and the same disaggregation yield as ΔNClpB (Figure 5a,b). These results are consistent with the reported effects of similar mutations in the full-length ClpB^13^ and in ΔNClpB from *E. coli*.^53^ The FRET efficiency histogram of the hyper-ΔNClpB variant was shifted to higher values compared to ΔNClpB data (Figure 5c). The transition rates of the MD, extracted from the analysis of smFRET trajectories of this mutant, were altered (Table S5), *k*_12_ = 4100 ± 400 s^–1^ and *k*_21_ = 7800 ± 400 s^–1^, resulting in the relative populations of the active and inactive states of 0.65 ± 0.01 and 0.35 ± 0.01. Consequently, the active/inactive state ratio was 1.85 ± 0.07 (Table S4), higher than in ΔNClpB, and similar to the result for the activated mutant of the full-length protein.^13^

Point-mutation E283A within the MD (repr-ΔNClpB, corresponding to E423A in the full-length ClpB) stabilizes its contacts with NBD1 and with MDs of neighboring subunits.^22,51,54,55^ Repr-ΔNClpB had a slightly decreased (by 10%) ATPase activity compared to ΔNClpB. Its disaggregation yield was reduced by half compared to the nonmutated ΔNClpB (Figure 5a,b), and the rate of disaggregation was significantly lowered (0.05 ± 0.1 nM.min^–1^), in good agreement with the previously observed effects of this mutation in the full-length protein.^13,54,55^ The FRET efficiency histogram of repr-ΔNClpB was shifted to lower average values in comparison to ΔNClpB. The derived interconversion rates were *k*_12_ = 5500 ± 150 s^–1^ and *k*_21_ = 4200 ± 300 s^–1^ (Table S5), leading to an increased relative population of the inactive state (0.57 ± 0.04) and the active/inactive state ratio of 0.76 ± 0.07, in agreement with the result of this mutation in the full-length ClpB (Table S4).^13^ Therefore, both the activating and repressing mutations strongly affected the MD dynamics of ΔNClpB. This suggests that the salt bridges between the MD and NBD1 that regulate the MD conformations in ClpB remain unaffected by NTD deletion.

We also probed another previously characterized pathway of MD regulation that involves the nucleotide binding sites of ClpB. Recently, we found that ATP binding to NBD1 decreased the population of the active state of the MD, whereas ATP binding to NBD2 had the opposite effect.^13^ We set to determine whether this regulation was retained in ΔNClpB. To this end, we generated and analyzed mutants of the Walker A motifs, which are located in the two NBDs and are responsible for ATP binding (Figure S8).^56^ We found no difference in their activity or in their MD dynamics in comparison to the corresponding full-length ClpB mutants,^13^ suggesting that NTD removal does not affect the allosteric regulation of the MD conformations upon nucleotide binding.

## Discussion

**Figure 6:**
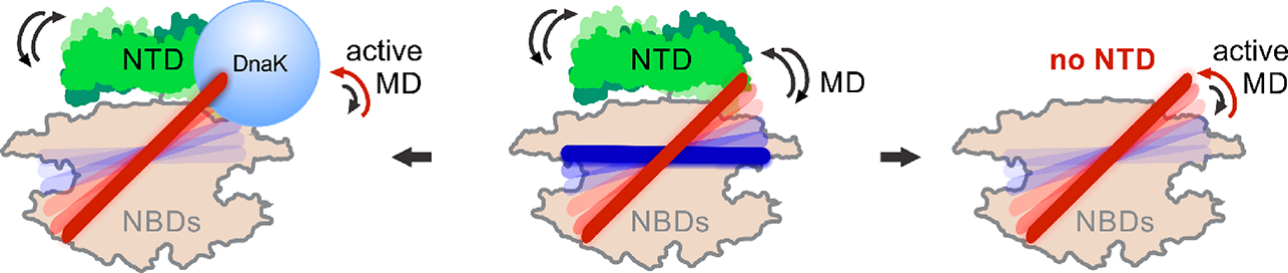
Schematic summary of entropic inhibition in ClpB by its NTD. Center: In the full-length ClpB, the NTD (green) and the MD undergo microsecond-time-scale motions (shown by reversible arrows). The MD is in dynamic equilibrium between active/inactive states (red/dark blue) with a state population ratio of ∼1. The NTD limits the conformational space of the MD by exerting a steric exclusion effect through its ultrafast movements. This in turn leads to autoinhibition of ATPase and disaggregation activities of ClpB but does not preclude potential DnaK binding to the MD. Left: DnaK binding to the MD (light blue, not drawn to scale) leads to a higher population of the active state of the MD, which in turn activates ClpB, as we reported in our previous study.^13^ Right: In the ΔNClpB variant, which lacks the NTD, the MD suppression by the NTD is lost, and it is free to sample its full conformational space. This also results in a more populated active state of the MD, with an increase of the active/inactive state ratio to ∼1.3, and an overall activation of ClpB even in the absence of DnaK. In bacterial cells, >20% of the molecules of ClpB lack the NTD domain and may contribute around a third of the overall thermotolerance,^18^ making this variant of physiological importance.

In this work, we studied the dynamics of the NTD and its communication with other domains of ClpB using a powerful combination of smFRET experiments, photon-by-photon H^2^MM analysis, fluorescence quenching measurements, and biochemical assays. We found that the NTD is involved in a novel mechanism of autoinhibition. This finding is unexpected: while the regulatory role of the MD was firmly established by multiple previous studies to act as the main regulatory switch of ClpB’s activity,^13,22,49,51,57^ the NTD is often viewed as a nonessential substrate-docking domain.^32^ Indeed, through its microsecond-time-scale dynamics, the NTD is able to exert an internal volume exclusion effect that significantly restricts the conformational space of the regulatory MD of ClpB and suppresses its tilting. This new mode of inhibition through conformational fluctuations, which we term *entropic inhibition*, directly depends on the fast motions of the NTD in and out of the conformational space of the MD, does not require strong interactions, and ultimately leads to inhibited bulk activities of ClpB. More specifically, our fluorescence quenching data showed that α-helix A1 of the NTD makes steric contacts with motif 2 of the MD. The apparent restriction of the MD motions by the NTD results in the suppression of ATPase activity and the inhibition of the disaggregation by ClpB (Figure 6). We directly verified the importance of the steric effect of the NTD on the MD by observing that NTD removal resulted in tilting of the MD in ClpB, shifting its active/inactive population ratio from 1 to 1.3, which in turn activated the machine and increased both its ATPase and disaggregation activities.

The entropic inhibition of ClpB by its NTD involves the functionally important motif 2 of the MD. This motif comprises the DnaK-binding epitope of ClpB,^49^ essential to its disaggregation function.^22,49,57^ Importantly, the fast movements of the NTD facilitate the approach of a DnaK molecule toward the MD and its binding,^49^ which removes the inhibition and can cause the release of bound substrate–proteins.^58^ However, as long as a DnaK molecule has not bound to the MD, the overall activity level of the machine remains low. This yet unrecognized role of the NTD as a regulatory element for ClpB’s inactivation is depicted in Figure 6. The NTD operates through its physical interaction with the major regulatory MD and prevents ClpB’s futile activity in the absence of substrate–protein and DnaK binding.

Previous studies have established the substrate-binding properties of the NTDs.^19,20,28,29,59^ It was found that the NTD is crucial for the binding of large protein aggregates that would otherwise not be recognized by ClpB^28^ and that the NTD of Hsp104 could facilitate dissolution of prions, which was not possible in the Hsp104 lacking the NTD.^59^ Therefore, the NTD has a dual function. In the basal state, in the absence of substrate–protein binding, the NTD serves to limit the ATPase and the disaggregation activities of the machine by the entropic inhibition mechanism. Upon binding of specific aggregated protein substrates by the NTDs, the inhibition is released and thus the machine activity is initiated.

Notably, our data suggest that the NTDs slightly reorient themselves with respect to NBD1 upon substrate–protein binding becoming closer to the central channel. This behavior is to be contrasted with p97, mentioned above, whose NTDs undergo a large upward movement with out-of-plane rotation of 75° upon activation.^60^ The presence of these conformational changes in the NTD upon substrate–protein binding is in agreement with recent cryo-EM structure of ClpB, where a trimer of substrate-bound NTDs from alternating protomers was shown to form a ring at the channel entrance, maintaining the overall helical arrangement of the protomers.^26^ Nevertheless, the NTD actually moves faster by ∼50% upon substrate binding, which might have an effect on the dynamics of DnaK binding to the MD, thereby influencing the activation state of the whole machine. To note, an AAA+ protein ClpA, which is structurally very similar to ClpB, has an almost identical NTD^14^ but no MDs. The addition by evolution of an MD in ClpB may have allowed the incorporation of an additional layer of control that involves autoinhibition by the NTD and activation by DnaK binding.

Consistent with the autoinhibitory role of the NTD, ΔNClpB is an activated ClpB variant that has tilted MDs and elevated basal ATP hydrolysis rate, and shows high disaggregation activity toward heat-induced aggregates of G6PDH and firefly luciferase. The increased basal ATP activity exhibited by ΔNClpB in comparison to the full-length variant is in good agreement with multiple preceding studies^17,19,20,31−36^ and implies that this truncated mutant of ClpB is more wasteful in terms of ATP consumption in the absence of bound substrate–proteins. Our data show unaltered yield and a faster rate of disaggregation following the NTD removal. Thus, we can conclude that ΔNClpB is a more active disaggregase, at least toward certain protein substrates. The presence of a small fraction of the ΔNClpB variant, with its high intrinsic ATPase activity and increased disaggregation activity, might be crucial for cell survival under conditions of particularly harsh heat shock and stress.

We conclude that the NTD is involved both in substrate binding and in the crucial autoinhibition of machine activation through the mechanism of *entropic inhibition*. Thus, the conformational fluctuations of the NTD prevent the allosteric switch of ClpB, the MD, from activating the machine. The NTD is absent in a significant fraction of naturally synthesized bacterial ClpB molecules, which lack this autoinhibition. Even more importantly, the fast motions of the NTD do not preclude the binding of DnaK to the MD, which also rescues it from the partially inhibited state, generating a fully active machine. A similar multimodal allosteric control through flexibly connected and fast-moving domains is likely to be present in numerous AAA+ proteins and other multidomain molecular machines to enable their rapid regulation under changing cellular conditions.

## Methods

### Protein Expression and Purification

*Thermus thermophilus* ClpB (*TT*. ClpB); its ΔNClpB variant beginning at residue 141 (Val); its mutants (Figure S3), cochaperones DnaK, DnaJ, and GrpE, cloned into a pET28b vector; and the isolated N-terminal domain of ClpB (residues 1–141), cloned into pProEx vector, were expressed and purified according to recently published protocols,^13^ as detailed in the Supplementary Methods.

### ATP Activity Measurements

The ATP activity of ClpB variants was measured using a coupled colorimetic assay.^61^ ClpB or its mutants (1 μM total monomer concentration) were incubated with 2 mM ATP and an ATP regeneration system (2.5 mM phosphoenol pyruvate, 10 units/ml pyruvate kinase, 15 units/ml lactate dehydrogenase, 2 mM 1,4-dithioerythritol, 2 mM EDTA, 0.25 mM NADH) in 50 mM HEPES (pH 8), 50 mM KCl, and 0.01% Tween 20. For the experiments in the presence of the model substrate κ-casein (Sigma-Aldrich), it was added to a final concentration of 50 μM. ATP hydrolysis was initiated by the addition of MgCl_2_ (10 mM) and measured by monitoring the time-dependent decrease in NADH absorption at 340 nm at 25 °C. Data were background-corrected in all cases, and the measured ATP hydrolysis rate per ClpB monomer per minute is presented.

### Disaggregation of Heat-Induced Aggregates of G6PDH and Luciferase

Disaggregation experiments were performed following previously described procedures.^13,46,62^ The protocol for aggregate preparation and disaggregation reactions is detailed in the Supplementary Methods.

### Labeling of ClpB and ΔNClpB Variants

Labeling of 23C-176C mutant of ClpB, S288C–S631C ΔNClpB, and any of its mutational variants was performed similarly to the previously reported protocol.^13^ First, the double-cysteine mutant of ClpB (or ΔNClpB) was incubated for 1 h with a 1:1.2 molar ratio of Alexa Fluor (AF) 594 (C5 maleimide, Invitrogen) under native conditions (25 mM HEPES, 25 mM KCl, pH 7). The unreacted dye was removed on a desalting column (Sephadex G25, GE Healthcare). The protein was exchanged into buffer containing guanidinium chloride in order to fully expose unlabeled cysteine residues (25 mM HEPES, 25 mM KCl, 2 M GdmCl, pH 7) and incubated with a 1:1.5 molar ratio of AF488 (C5 maleimide, Invitrogen) for 1 h to achieve complete labeling of unreacted cysteines, followed by the separation of unreacted dye on a desalting column. Labeling was confirmed by absorption measurements, and the fraction of the double-labeled AF488/594 species was consistently >30% as directly calculated from single-molecule measurements. Labeling of the full-length 487C-ClpB variants with Atto 655-maleimide (Sigma-Aldrich) was carried out by incubating ClpB with the dye at a 1:1.5 ratio for 5 h in the dark (25 mM HEPES, 25 mM KCl, 2 M GdmCl, pH 7), followed by the separation of unreacted dye on a desalting column (Sephadex G25, GE Healthcare), buffer-exchange to the native buffer (25 mM HEPES, 25 mM KCl, pH 7), and filtration of the labeled protein through a 0.22 μm filter (Millex, Millipore).

### Mixing of Fluorescently Labeled and Unlabeled Protomers

For the preparation of ClpB and ΔNClpB assemblies for smFRET experiments, AF488-AF594-labeled double-cysteine mutants were combined with a 100-fold molar excess of unlabeled cysteine-less ClpB or ΔNClpB, respectively. This ratio ensured that the probability of the incorporation of one labeled protomer in a hexamer was 5.7%, whereas the probability to find two labeled protomers in the same hexamer was as low as 0.15%. To achieve full mixing, the protein solutions were initially dialyzed in the presence of 6 M GdmCl. This was followed by dialysis steps in the presence of 4, 2, 1, and 0 M GdmCl. The final steps involved extensive dialysis into a low-salt buffer (25 mM HEPES, 25 mM KCl, 10 mM MgCl_2_, 2 mM ATP, pH 8) and filtration through 0.1 μm filters (Whatman Anotop-10). The assembled ΔNClpB was aliquoted, flash-frozen, and stored at −80 °C until further use. For the preparation of activated, repressed ΔNClpB and its Walker A mutant hexamers, fluorescently double-labeled ΔNClpB bearing the respective mutation was mixed with the 100-fold excess of unlabeled ΔNClpB with the same mutation but without cysteine residues, and dialyzed according to the above protocol. For the preparation of ΔHA1ClpB hexamers of ClpB, double-labeled ΔHA1ClpB was mixed with the ΔHA1ClpB mutant of ClpB without cysteine residues. These labeling and mixing procedures result in correctly folded protein, as shown in our previous study,^13^ and as can be inferred from the ATPase and disaggregation activities, which are similar to those of the wild-type protein.

### Single-Molecule Measurements

The preparation of custom-made glass flow chambers for single-molecule experiments was carried out following previously reported protocols^41,44,63^ and is detailed in the Supplementary Methods. The chambers were coated with a supported lipid bilayer composed of egg phosphatidylcholine (Avanti Polar Lipids) to prevent protein absorption during measurements. The assembled hexamers of ClpB (or ΔNClpB) were diluted to ∼50 pM of labeled ClpB, corresponding to ∼5 nM of total ClpB, into a single-molecule buffer (25 mM HEPES, 25 mM KCl, 10 mM MgCl_2_, 2 mM ATP, 0.01% Tween 20, pH 8). The solutions were loaded into the chambers, which were rapidly sealed to prevent evaporation. Measurements were conducted using a home-built inverted confocal single-molecule microscope,^41,44,63^ which is described in detail in the Supplementary Methods. Data collection was carried out on freely diffusing molecules as reported.^13^ Briefly, the samples were illuminated with focused and overlapped 485 and 594 nm diode laser beams pulsed at a ratio of 3:1 with a repetition rate of 40 MHz. The emitted photons were divided into two channels using a dichroic mirror (FF580-FDi01, Semrock) and passed through band-pass filters, ET-535/70m for the AF488 emission and ET-645/75m for the AF594 emission (Chroma). The arrival times of the emitted photons in both channels were registered by single-photon avalanche photodiodes (PerkinElmer SPCM-AQR-15) coupled to a standalone time-correlated single photon counting module (HydraHarp 400, PicoQuant). Data were acquired for up to 3.5 h per sample at ambient temperature (22 °C). Between two and four individual samples were prepared and measured for each ClpB variant and condition.

### smFRET Data Analysis

Data analysis was performed as recently described.^13^ A cutoff of 10 μs was used to effectively separate fluorescence bursts from the background. FRET efficiency and stoichiometry were calculated as detailed elsewhere.^64^ We applied several corrections to eliminate any artifacts and to ensure that only fluorescent photons arising from individual molecules entered the analysis. More details on the data selection can be found in the Supporting Information section, Figure S4. FRET efficiency values were corrected for the leakage of photons from the donor to acceptor channel (estimated to be no more than 7%). Using stoichiometry/FRET efficiency histograms (Figure S4), any singly labeled species were eliminated, and only the molecules containing both AF488 and AF594 were selected for further analysis. For the photon-by-photon H^2^MM analysis, fully detailed previously,^41^ the photons arising from preselected molecules after donor-only excitation were used. A fixed number of molecules (5800) was analyzed for each sample. The data were fitted with a three-state model. In the case of the analysis of MD dynamics, relative populations of the two major states were extracted and compared between different ΔNClpB mutants or conditions (Table S4). This fitting approach was rigorously tested and confirmed through multiple additional methods on the equivalent full-length ClpB data sets in our preceding study.^13^

### FLCS Measurements

Conventional smFRET measurements of freely diffusing double-labeled molecules of 23C-176C hexamers (assembled in 1:100 ratio with WT ClpB) were performed. Pulsed interleaved excitation by 485 and 594 nm lasers was used (in 1:1 sequence). The measurements were conducted with laser powers of 50 μW (485 nm) and 10 μW (594 nm), a repetition rate of 40 MHz and a TCSPC time resolution of 16 ps. Fluorescence lifetime components were extracted from the low FRET efficiency (below 0.18) and the high FRET efficiency (above 0.75) populations. Photon weights were calculated for the two lifetime components (low FRET and high FRET population), according to Kapusta et al.^43^ Subsequently, the same ClpB samples were measured at 1 nM, with pulsed excitation by the 485 nm laser at 50 μW, 40 MHz, and a TCSPC time resolution of 16 ps. Filtered FCS cross-correlation functions were calculated using the photon weights of the low FRET and high FRET populations as previously described.^43^ The filtered cross-correlation curves were fitted to the equation (Origin Pro 2019):
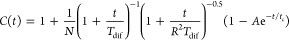
1where *N* is the number of molecules in the confocal volume, *T*_dif_ is the diffusion time, *R* is the ratio of the dimensions of the Gaussian-shaped beam waist parallel and perpendicular to the direction of light propagation, *A* is an amplitude, and *t*_c_ is the correlation time. The value of *R* was kept constant (at 6.2) for the fitting and was determined from the FCS calibration measurement using a reference Rhodamine 6G solution with a manufacturer-provided diffusion coefficient (3.84 × 10^–6^ cm^2^ s^–1^ in water at 22.4 °C). The results are shown in Figure 2c.

### Rotational Simulation of the NTD

To obtain further information on the possible interactions of the NTD with the MD, we simulated multiple conformations of the NTD around its linker and mapped out possible interactions with the MD. First, we used the structural model of *TT* ClpB^12^ to create multiple conformations of the NTD by rotating it as a rigid body around a point within the NTD-NBD1 linker (residue 142). We then excluded all conformations that caused a steric clash within the same protomer or with adjacent protomers. In our case, a clash was defined as a van der Waals (VDW) contact between the MD atoms and the other atoms of the ClpB hexameric complex (eq 2):
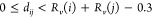
2where *R*_*v*_(*i*) and *R*_*v*_(*j*) are the VDW radii of atoms *i* and *j*, respectively, and *d*_*ij*_ is the distance between the two atoms. In eq 2, 0.3 Å is our clash threshold value (lower bound for a VDW contact), which was calculated from the structure of the hexameric model.^30^ We excluded all conformations that had at least one distance within the ranges calculated using eq 2. We then calculated the distances between all the NTD residues and residue 487 on the MD. A threshold of 10 Å was used to select NTD structures within this range of distances. We found that α-helix A1 of the NTD was in close proximity to the MD (Figure S3).

### Fluorescence Measurements of Atto 655-Labeled ClpB

Steady-state fluorescence of Atto 655 labeled ClpB mutants (either with no additional tryptophans (Ws), 12W or 23W) was measured using a Fluorolog-3 spectrofluorometer (Horiba Jobin Yvon, Edison, NJ, USA). The excitation wavelength was set to 635 nm, and fluorescence was collected between 650 and 800 nm. Measurements were conducted using 0.5 μM solutions of Atto 655-labeled ClpB mutants in 25 mM HEPES, 25 mM KCl, 10 mM MgCl_2_, and 2 mM ATP, at pH 8 and at ambient temperature (25 °C). All spectra were collected in duplicate per mutant and background-corrected. Integrated average fluorescence values are listed in Table S6.

### Ensemble Fluorescence Lifetime Measurements

Fluorescence lifetime measurements were performed on a FluoroHub time-correlated single-photon counting instrument (Jobin-Yvon), by using a time-correlated single-photon counting method.^48^ 50 nM solutions of Atto 655-ClpB mutants, prefiltered through 0.1 μm filters (Whatman Anotop-10), were measured (in 25 mM HEPES, 25 mM KCl, 10 mM MgCl_2_, 2 mM ATP, pH 8), with four repeats per sample. A pulsed diode laser with an emission at 635 nm was used as an excitation source, with a pulse length of 360 ps (FWHM). The emission was collected at 680 nm, with a slit width of 12 nm. Photons were collected in 2048 channels, with a peak maximum of 10 000 counts. Data were globally fitted with a two-exponential model using a built-in fitting wizard, and average fluorescence lifetimes are reported (Table S7).
